# The Charleston Medical College

**Published:** 1859-04

**Authors:** 


#### THE CHARLESTON MEDICAL COLLEGE.

An annual catalogue of the South Carolina Medical College at Charleston, has just been received, and is before us. This excellent institution has long and deservedly maintained a high and honorable position among the medical colleges of the land. The unquestionable professional merit, dignified bearing, and courteous and generous spirit which so signally characterize its able Faculty, cannot but secure for it a permanent place in the confidence of the public mind. With all the discouraging rumors to which the prevailing epidemic gave rise, for a short time anterior to the opening of the last session, we are glad to see by the record, that 195 students were in attendance.

It was a subject of regret, however, to notice the loss which the institution has sustained—present and prospective—in the person of (the lamented successor of Dr. Dickson,) Prof. P. C. Gaillard. His high grade of mind—his ample and accurate medical attainments, and his noble independence of character, blended with a fascinating suavity of manners, won early laurels for his brow, and attracted a large circle of appreciating friends. It must, however, be highly gratifying to the friends of the college, to learn that the chasm so unexpectedly made in the Board of Instruction, has been so promptly and ably filled by the election of the distinguished gentleman who so long and ably occupied the Chair of Surgery in the same Institution, viz., Dr. E. Geddings. The college still retains our kindest wishes for its continued success.

